# *QuickStats:* Age-Adjusted Death Rates* for Four Selected Mechanisms of Injury^†^ — National Vital Statistics System, United States, 1979–2019^§^

**DOI:** 10.15585/mmwr.mm7020a4

**Published:** 2021-05-21

**Figure:**
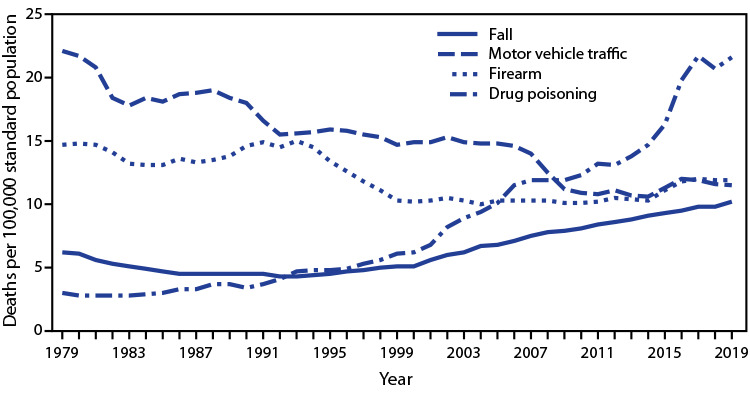
In 1979, of the four mechanisms of injury, age-adjusted mortality rates were highest for motor vehicle traffic deaths and lowest for drug poisoning deaths. From 1979 to 2019, the age-adjusted rate of motor vehicle traffic deaths decreased from 22.1 per 100,000 to 11.1, and the rate of firearm-related deaths decreased from 14.7 to 11.9. During the same period, the rate of drug poisoning (overdose) deaths increased from 3.0 to 21.6, and the rate of fall-related deaths increased from 6.2 to 10.1. In 2019, the rates were highest for drug poisoning deaths and lowest for fall-related deaths.

**Source:** National Vital Statistics System compressed mortality file, underlying cause of death. https://wonder.cdc.gov/mortsql.html

For more information on these topics, CDC recommends the following link: https://www.cdc.gov/injury

